# Mutated RAS-associating proteins and ERK activation in relapse/refractory diffuse large B cell lymphoma

**DOI:** 10.1038/s41598-021-04736-0

**Published:** 2022-01-17

**Authors:** Alexandre Benoit, Elisabeth Bou-Petit, Hsiang Chou, Melissa Lu, Cynthia Guilbert, Vincent Mingyi Luo, Sarit Assouline, Ryan D. Morin, Svetlana Dmitrienko, Roger Estrada-Tejedor, Nathalie A. Johnson, Koren K. Mann

**Affiliations:** 1 Lady Davis Institute, Jewish General Hospital, 3755 Côte Sainte-Catherine Road, Montreal, QC H3T 1E2 Canada; 2 Division of Experimental Medicine, McGill University, Montreal, QC Canada; 3 Grup de Química Farmacèutica, IQS School of Engineering, Universitat Ramon Llull, Barcelona, Spain; 4 Université de Montréal-Faculté de Médecine, Montreal, Canada; 5 Department of Microbiology and Immunology, McGill University, Montreal, QC Canada; 6 Gerald Bronfman Department of Oncology, McGill University, Montreal, QC Canada; 7 Department of Molecular Biology and Biochemistry, Simon Fraser University, Burnaby, BC Canada; 8 Division of Pathology, McGill University Health Centre, Montreal, QC Canada

**Keywords:** B-cell lymphoma, Cancer therapeutic resistance

## Abstract

Diffuse large B cell lymphoma (DLBCL) is successfully treated with combination immuno-chemotherapy, but relapse with resistant disease occurs in ~ 40% of patients. However, little is known regarding relapsed/refractory DLBCL (rrDLBCL) genetics and alternative therapies. Based on findings from other tumors, we hypothesized that RAS-MEK-ERK signaling would be upregulated in resistant tumors, potentially correlating with mutations in RAS, RAF, or associated proteins. We analyzed mutations and phospho-ERK levels in tumor samples from rrDLBCL patients. Unlike other tumor types, rrDLBCL is not mutated in any Ras or Raf family members, despite having increased expression of p-ERK. In paired biopsies comparing diagnostic and relapsed specimens, 33% of tumors gained p-ERK expression, suggesting a role in promoting survival. We did find mutations in several Ras-associating proteins, including GEFs, GAPs, and downstream effectors that could account for increased ERK activation. We further investigated mutations in one such protein, RASGRP4. In silico modeling indicated an increased interaction between H-Ras and mutant RASGRP4. In cell lines, mutant RASGRP4 increased basal p-ERK expression and lead to a growth advantage in colony forming assays when challenged with doxorubicin. Relapsed/refractory DLBCL is often associated with increased survival signals downstream of ERK, potentially corresponding with mutations in protein controlling RAS/MEK/ERK signaling.

## Introduction

Diffuse large B cell lymphoma (DLBCL), the most common non-Hodgkin Lymphoma, is a heterogenous disease, with several gene expression and mutational signatures identified^1^. Germinal center B-cell (GCB) and activated B-cell (ABC) are the two major molecular subtypes of DLBCL, arising from different steps in B cell activation, leading to the distinct signatures and clinical outcomes. Further refinement of the classifications uses both mutations and gene expression^2^. However, all patients with DLBCL currently receive the same treatment, R-CHOP; rituximab, cyclophosphamide, doxorubicin, vincristine, and prednisone^3^. Despite significant efficacy, DLBCL relapses in approximately 40% of the patients with chemotherapy-resistant disease, which is fatal in 90% of patients^4^.

R-CHOP resistance mechanisms in vivo are unclear, however several means have been postulated^5^. These include downregulation of the target for rituximab, CD20, co-expression of MYC and BCL-2, overexpression of other anti-apoptotic proteins, and sustained survival signals from NF-κB and ERK^6–8^. *TP53* mutation is associated with a poor prognosis in DLBCL, and these mutations are enriched in multiple relapsed/refractory DLBCL cohorts^9–12^. However, many of these data were generated in cell line models or in retrospective, correlative studies of fixed tissue.

The canonical RAS-RAF-MEK-ERK pathway promotes resistance to chemotherapy in many tumor types^13–15^. The RAS subfamily consists of 3 small GTPases, namely H-Ras, K-Ras, and N-Ras^16^. RAS proteins are turned “on” when guanyl exchange factors (GEF) exchange GDP to GTP, which activates RAS. GTPase activating proteins (GAP) promote the GTPase activity of Ras, which will lead to its inhibition. GTP-bound RAS activates downstream MAPK signaling (the RAF/MEK/ERK kinase cascade). In addition, PI3K can also been stimulated by activated RAS, through binding the p110 PI3K subunit, with subsequent target phosphorylation (i.e. AKT). Ras signaling is then propagated by downstream effectors, leading to proliferation, differentiation, migration, and adhesion. *RAS* is frequently mutated in cancer, where activating mutations occur in at least 15% of all cancers, leading to oncogenic phenotypes^16,17^. In addition, *RAF* genes are frequently mutated, including the *BRAF* gene, where the V600E mutation is successfully targeted by the drug vemurafenib in melanoma^18^ and has been identified and successfully targeted in hairy cell leukemia (a B cell malignancy)^19,20^.

Based on the importance of RAS/MEK/ERK activation in other resistant tumors, we predicted activation of this pathway in relapsed/refractory DLBCL (rrDLBCL). Indeed, we observed that compared to samples at diagnosis, ERK activation is gained in 33% of rrDLBCL samples, but no mutation in any *RAS* or *RAF* genes^10^. However, we have identified mutations in several RAS-associated proteins, including proteins playing a role in proliferation, survival, apoptosis, adhesion and migration. We hypothesized that mutations in RAS-associating proteins give a gain of function to GEFs and effectors or a loss of function to GAPs, which will result in enhanced downstream signaling and resistance to R-CHOP. We further explored one candidate GEF, RAS Guanyl Releasing Protein 4 (RASGRP4), which was previously shown to play an oncogenic role in DLBCL^21^. While no RASGRP4 mutations were identified in this study, we show that RASGRP4 mutations observed in rrDLBCL patient samples enhanced p-ERK activation, as well as increased resistance to doxorubicin.

## Results

### Phospho-ERK levels are increased in rrDLBCL patients despite lack of RAS/RAF mutations

**Figure 1:**
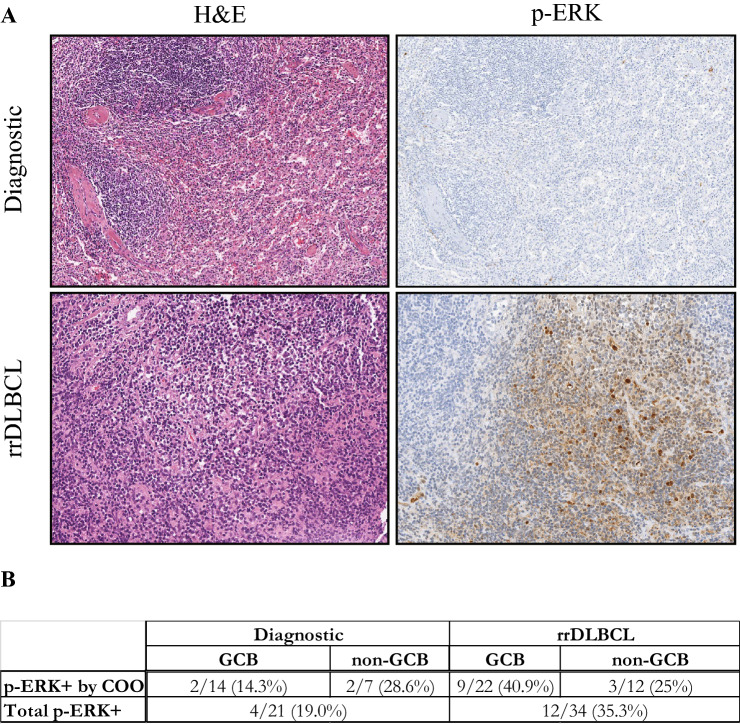
(**A**) Representative images of p-ERK in matched pairs of diagnostic and rrDLBCL samples. (**B**) Gain of phosphorylation of ERK in rrDLBCL tissues compared to diagnostic DLBCL tissues, including matched paired biopsies.

We hypothesized that, like many tumor types, RAS/MEK/ERK pathway activation could be a resistance mechanism and thus, activity would be found with greater prevalence in rrDLBCL as compared to diagnosis. Canonical MAPK activation is associated with increased RAS activation, and subsequent increased ERK activity, as detected by ERK1/2 phosphorylation (p-ERK) on residues T202/Y204. We stained DLBCL patient samples containing 21 matched diagnostic and relapse biopsies, as well as 13 additional rrDLBCL samples. We found that 35% of the relapse samples were p-ERK positive compared to 19% in diagnostic samples (*p* = 0.007) (Fig. 1). Importantly, 33% of the paired biopsies gained p-ERK staining at relapse. Interrogating the MAPK pathway activation status within DLBCL subtypes, we noted that a remarkable 75% of the positive p-ERK rrDLBCL samples were GCB-DLBCL (*p* = 0.0015).

We hypothesized that activation of this pathway might be due to a higher prevalence of activating *RAS* or *RAF* mutations. We interrogated our previously published data on a patient cohort that included both rrDLBCL and relapse/refractory TLy (rrTLy), which is are rapid-growing tumors sharing several histological features with DLBCL patients^10^. Surprisingly, no mutations in any of the three *RAS* genes were found, despite the prevalence of *RAS* mutations in many other tumor types. In a query of 5 datasets from de novo DLBCL tumors (total of 1295 patient samples), the prevalence of *RAS* mutations was also very low at diagnosis: 24 of 1295 (1.9%) for *KRAS*, 10 of 1295 (0.8%) for *HRAS*, and 1 of 1295 (0.1%) for *NRAS*^2,22–25^. Also, no *RAF* genes or p110α, δ, and γ isoforms of Class 1 PI3K were mutated. We found one mutation in p110β but it is the only catalytic subunit of the Class 1 PI3K shown to not interact with RAS^26^. Thus, the increase in p-ERK staining at relapse could not be attributed to a gain of *RAS* or *RAF* mutations.

### RAS-associating protein mutations in relapse/refractory DLBCL

**Figure 2:**
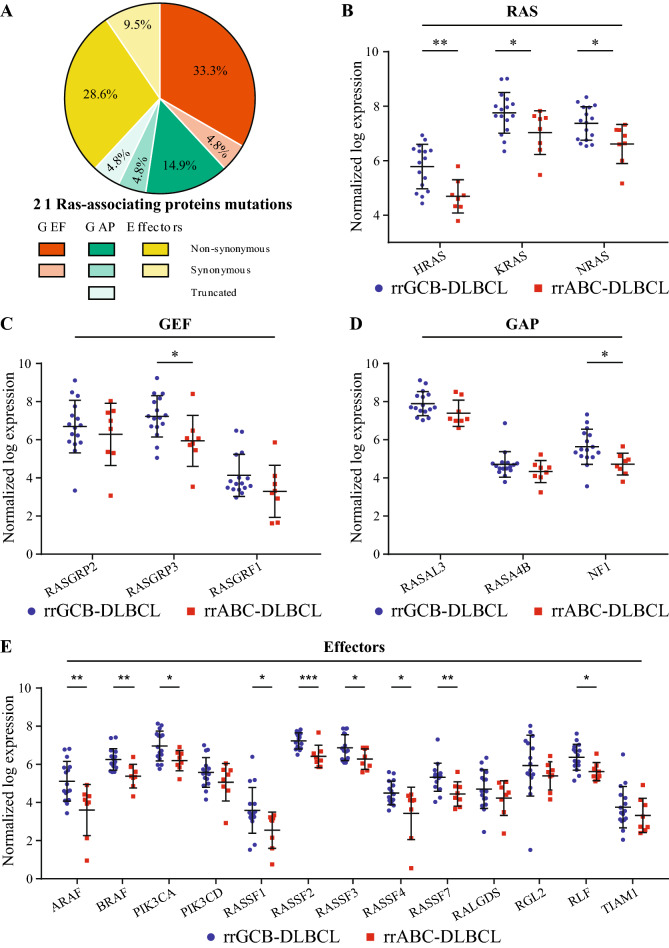
(**A**) Proportion of non-synonymous, synonymous and truncated mutations in each type of RAS-associating proteins. (**B**) RAS mRNA expression level is higher in rrGCB-DLBCL samples compared to rrABC-DLBCL samples. GEF (**C**), GAP (**D**), and Effector (**E**) mRNA expression level is higher in rrGCB-DLBCL samples compared to rrABC-DLBCL samples. (unpaired t-test; * = *p* < 0.05, ** = *p* < 0.01, *** = *p* < 0.001).

During mutational analysis, we found 14 of 39 samples (36%) harbored a mutation in a subset of genes coding for RAS-associating protein (Table 1): including GEFs (40%), GAPs (25%), and effectors (35%) (Fig. 2A). We found 20 mutations in 16 different genes encoding for a RAS-associating protein in the rrDLBCL samples (Table 1 and Supplemental Fig. S1). Of the 14 samples mutated, 5 samples (36%) had more than one RAS-associating protein gene mutated: 4 samples had 2 genes mutated and 1 sample had 3 genes mutated. Most of these mutations were missense (75%), with only one non-sense mutation in a GAP. More than 1/3 of the mutations are predicted to affect the protein structure and function using the PolyPhen-2 software (Table 1)^27^. Twenty-seven percent of the rrDLBCL samples (7/26) and 54% of the rrTLy samples (7/13) had a mutation in a RAS-associating protein. Of the 1295 combined samples of diagnostic DLCBL queried, none of genes was mutated at a rate higher than 1%, except NF1 (3%; Supplementary Table 1)^2,22–25^.

**Table 1:** In rrDLBCL and rrTLy samples, 20 mutations were found in 16 different genes encoding for a RAS-associating protein.

Type of RAS-associating protein	Gene	Mutation type	Annotation/effect	COO	Pathology	PolyPhen-2	
						Score	Prediction
GEF	RASGRF1	Missense	L392P	GCB	DLBCL	0.999	Probably damaging
	RASGRF1	Missense	T796K	GCB	TLy	1	Probably damaging
	RASGRF2	Missense	D172N	GCB	DLBCL	0.611	Possibly damaging
	RASGRP3	Missense	F70L	GCB	DLBCL	0	Benign
	RASGRP4	Missense	R280K	GCB	TLy	0	Benign
	RASGRP4	Silent	R304R	GCB	DLBCL	N/A	N/A
	RASGRP4	Missense	D404N	GCB	DLBCL	1	Probably damaging
	SOS1	Missense	V524F	ABC	DLBCL	0.113	Benign
GAP	NF1	Silent	L234L	GCB	TLy	N/A	N/A
	RASA2	Missense	Y325D	GCB	DLBCL	0.185	Benign
	RASA2	Nonsense	Q491*	GCB	DLBCL	N/A	N/A
	RASA4	Missense	D111N	GCB	TLy	1	Probably damaging
	RASAL2	Missense	A22V	GCB	TLy	0.221	Benign
Effector	PLCE1	Missense	W893R	GCB	TLy	0.016	Benign
	RALGDS	Missense	E644G	GCB	TLy	0.999	Probably damaging
	RASSF1	Missense	A192V	ABC	DLBCL	0.001	Benign
	RASSF2	Missense	G96V	GCB	TLy	0.947	Possibly damaging
	RASSF9	Silent	L93L	GCB	TLy	N/A	N/A
	RGL2	Missense	V428M	ABC	TLy	0.998	Probably damaging
	TIAM1	Silent	V434V	GCB	DLBCL	N/A	N/A

The possible impact of the missense mutations on the structure and the function of the proteins was predicted with PolyPhen-2 software version 2.2. PolyPhen-2 classified a missense mutation as Probably damaging (more confident prediction), Possibly damaging (lower confident prediction) as Benign based on a False Positive Rate^27^.

There was a more than twofold bias towards Ras-associating protein mutation in GCB (11/24; 46%) versus ABC (3/14; 21%). Further, the gene expression level of *RAS*, *RAF*, and RAS-associating proteins at relapse were higher in GCB-DLBCL compared to ABC-DLBCL (Fig. 2B–E, Supplemental Fig. S2A). However, there were no significant differences in the expression of these genes, when comparing samples with and without a mutation in a RAS-associating protein, with the exception of a slight decrease in PIK3CD in mutated samples (Supplemental Fig. S2B). Of note, in an RNA sequencing analysis of 23 paired diagnostic and relapsed DLBCL biopsies, none of the Ras-associating protein genes were amongst the 14 recurrent differentially expressed genes comparing de novo and relapsed disease^28^.

### Mutations in the GEF domain of RASGRP4 in rrGCB-DLBCL patients

Among the mutated RAS-associating protein genes, we identified *RASGRP4*, a GEF mutated only in rrGCB-DLBCL. RASGRP4 is expressed in mast cells, monocytes, granulocytes, and neutrophils^29–31^. It is also involved in the development of normal T lymphocytes, T cell leukemia and acute myeloid leukemia^30,32^. Recently, RASGRP4 was reported to be highly expressed but not mutated in a limited number of DLBCL (4–7 cases) and other B cell malignancies (5 each of follicular lymphoma and Burkitt lymphoma), and when compared to normal and activated B cells^21^. RASGRP family members show specificity for either RAS or RAP1^33^. In contrast to the other RASGRP family members, RASGRP4 preferentially activates HRAS, does not need to be phosphorylated, and is constitutively active, although activity can be promoted by diacylglycerol^30,33,34^.

**Figure 3:**
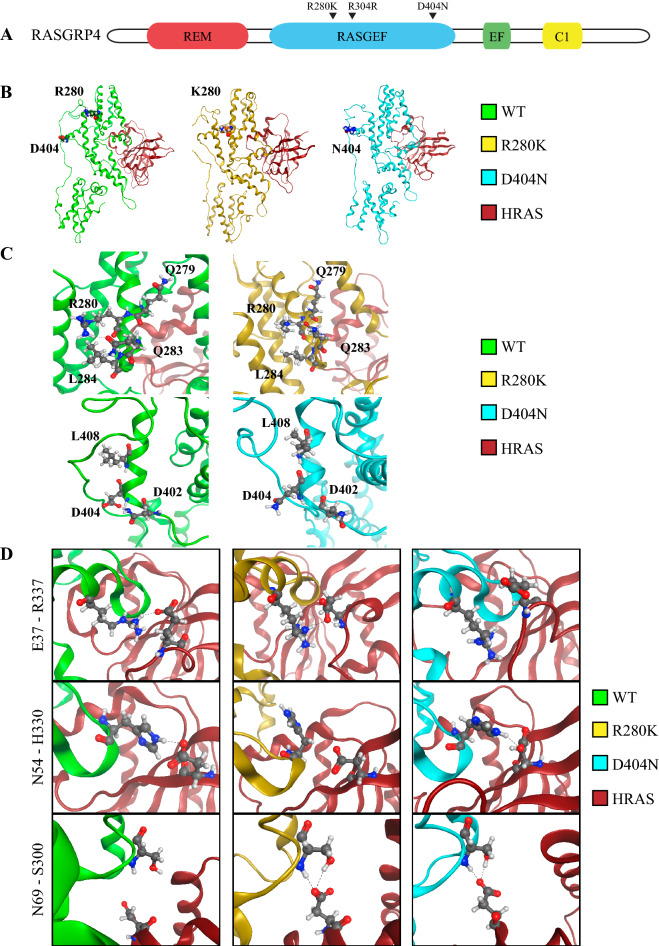
(**A**) Diagram on relevant domains within RASGRP4 with mutations highlighted. (**B**) Graphical representation of the three HRAS-RASGRP4 complexes predicted after applying molecular docking and molecular dynamics simulations. Mutations under study are highlighted. (**C**) Comparison of the intramolecular H-bond pattern established with the wild-type protein (left) and D404N and R280K mutated amino acids (right). (**D**) Changes on the intermolecular H-bond pattern between HRAS and RASGRP4 that could explain the different stabilities of the complexes. The first residue refers to HRAS and the second to RASGRP4.

*RASGRP4* was not mutated in our diagnostic samples or in 5 different DLBCL studies comprising a total of 1295 patients^2,22–25^. All *RASGRP4* mutations were localized in the GEF domain at position R280K, R304R and D404N (Fig. 3A). These mutations have not been reported previously, and no role has been identified for mutant RASGRP4 in DLBCL. Interestingly, when we compared the protein sequence of RASGRP4 to the other RASGRP family members, and between species, the amino acid at position D404 is conserved, while the amino acid at position R280 is not (Supplemental Fig. S3). Thus, the D404N mutation may result in a greater functional change than R280K (Table 1).

### In silico molecular dynamics simulation predicts increased binding of RASGRP4^D404N^ mutant to HRAS

To understand how the identified mutations in RASGRP4 alter the activity of this GEF, we examined the complexes between HRAS and the corresponding RASGRP4 mutants with amino acid changes by molecular dynamics (Supplemental Fig. S4). The resulting simulations were analyzed to estimate the binding free energy between HRAS and RASGRP4, a value representing the stability of the intermolecular interaction (Table 2). The differences between $\Delta G_{bind}$ of both mutations are statistically significant (*p* < 0.0001, n = 2000). The R280K mutant shows a moderate gain of the binding energy, while the presence of the D404N change increases the $\Delta G_{bind}$ about 33%. This indicates that more energy is required to disrupt the interactions between mutant RASGRP4 and HRAS and thus, are predicted to stabilize the interaction between HRAS and RASGRP4.

**Table 2:** Free energy of binding ($\Delta {\text{G}}_{{{\text{bind}}}}$) predicted for the interaction complexes involving HRAS and the three RASGRP4 systems considered (i.e. WT, R280K and D404N).

RASGRP4	$\Delta {\text{G}}_{{{\text{bind}}}}$	$\Delta \Delta {\text{G}}_{{{\text{bind}}}}$
WT	− 124.14 ± 13.53	–
R280K	− 133.60 ± 10.76	− 9.46 ± 24.29
D404N	− 165.39 ± 10.01	− 41.25 ± 23.54

The relative increase of the binding energy observed in RASGRP4 mutants compared to the wild-type protein are quantified as $\Delta \Delta {\text{G}}_{{{\text{bind}}}}$. All results are expressed as kcal/mol.

Of note, stabilization of the HRAS-RASGRP4 complex occurs even though the location of R280K and D404N mutations are not near the reported RASGRP4-HRAS-binding domain (Fig. 3B). Thus, we hypothesized that the mutation of these residues has a direct effect on the intramolecular H-bond pattern of RASGRP4 D404 or R280. The frequency at which either D404 or R280 form H-bonds with surrounding residues is measured during the simulation time and expressed as percent time (Fig. 3C). The RASGRP4 D404 residue of the wild type protein interacts 58% of the time with N402, an interaction that is broken in the presence of D404N mutation (Fig. 3C). In contrast, the interaction with L408 is maintained in both the wild-type and D404N mutated models. For the R280K model, the mutation changes the strength pattern of the H-bonds. On one hand, the interaction between R280 and Q283 is the most common H-bond of the wild type model (found 69% of the time), but it is dramatically reduced to 19% for the mutant (Fig. 3C). On the other hand, the interaction with L284 increases from 26 to 69% when mutating R280K. Only the interaction of R280 with Q279, which was already weak (10%), is broken on the mutated model.

The altered intramolecular H-bond pattern of RASGRP4 caused by the point mutations appear to induce differences in the intermolecular HRAS:RASGRP4 interaction patterns. Three pairs of residues could contribute to the enhanced stability of HRAS with mutant RASGRP4: E37-R337, D54-H330 and D69-S300, where the first residue refers to HRAS and the second to RASGRP4 (Fig. 3D). The interaction between E37 (HRAS) with R337 (RASGRP4) is present in the wild-type and R280K mutant models but lost in the D404N mutant. An H-bond between D54 (HRAS) and H330 (RASGRP4) occurs in the wild-type and the D404N mutation models but is lost in the R280K model. Importantly, a new H-bond between D69 (HRAS) and S300 (RASGRP4) is formed in both mutant models, which is not present in the wild-type model. Finally, two additional, slightly weaker interactions are present only in the mutant models: P34 and D54 from HRAS form two H-bonds with R337 and N331 from RASGRP4, respectively. Together, these data predict that mutant RASGRP4 interacts more tightly with HRAS through changes in the intramolecular organization of H-bonds with mutated RASGRP4 that could result in enhanced activation of the pathway.

### Activation of the RAS-MEK-ERK pathway in RASGRP4 mutant cells

**Figure 4:**
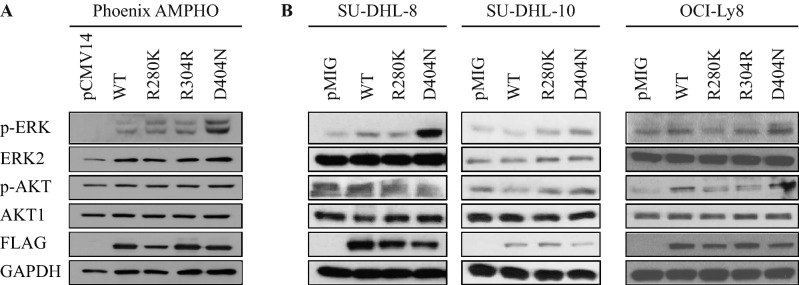
Mutant RASGRP4^D404N^ Phoenix AMPHO cells (**A**) and RASGRP4^D404N^ GCB-DLBCL (**B**) have a higher level of p-ERK than RASGRP4^WT^ cells in normal serum condition (10% FBS). AKT and p-AKT (Ser473) were not changed by mutational status.

Our in silico modeling of the RASGRP4^D404N^ mutation predicts a better stability of interaction with HRAS, which may result in increased p-ERK downstream of activated RAS, as observed in our patient cohort (Fig. 1). Thus, we wanted to test whether *RASGRP4* mutations would result in an increased activation of the RAS-MEK-ERK and AKT pathways in vitro by assessing p-ERK and p-AKT levels, respectively. First, we overexpressed mutated RASGRP4 transiently in the Phoenix Amphotropic (AMPHO) 293 T cell line, a model which does not have basal ERK1/2 constitutively phosphorylated, and a modest level of p-AKT, by western blotting (Fig. 4A). Of note, overexpression of RASGRP4, wild-type or mutant, increased both p-ERK and p-AKT levels. However, cells containing RASGRP4^D404N^ and RASGRP4^R280K^ express higher constitutive levels of p-ERK, the strongest being the D404N mutation. Interestingly, mutation of *RASGRP4* did not affect p-AKT levels. Cells expressing the silent mutation R304R have the same basal level of p-ERK as the wild-type RASGRP4.

To better model the lymphoma genetic background, we next created DLBCL cell lines expressing wild-type or mutant RASGRP4. We chose three GCB cell lines, because the RASGRP4 mutations were only detected in this DLBCL subtype. In contrast to the Phoenix-AMPHO, the basal level of p-ERK in the empty vector expressing cells was higher in the human GCB-DLBCL cell line OCI-Ly8, SU-DHL-8 and SU-DHL-10 (Fig. 4B). The cells overexpressing RASGRP4^WT^ have the same level of p-ERK as the empty vector except for the SU-DHL-8. Consistent across all GCB DLBCL cell lines and the Phoenix-AMPHO cells, overexpression of the RASGRP4^D404N^ mutant results in cells expressing a higher level of p-ERK than the empty vector- or wild-type RASGRP4-expressing cells. No significant changes in p-ERK levels were observed in cells expressing the RASGRP4^R280K^ mutation. Phospho-AKT levels were not enhanced when any cells expressed wild-type or mutant RASGRP4.

Next, we evaluated the induction of p-ERK following stimulation of mutant RASGRP4-expressing lymphoma cells using phorbol 12-myristate 13-acetate (PMA), a mimic of diacylglycerol. In mast cells, PMA activates RASGRP4 by inducing membrane localization, interaction with RAS, and subsequent ERK phosphorylation^35^. PMA induced p-ERK regardless of *RASGRP4* mutation, with the exception of OCI-Ly8 cells, where higher p-ERK levels were detected with the RASGRP4^D404N^ mutants (Supplemental Fig. S5A). We further investigated whether RASGRP4^D404N^-expressing cells had altered sensitivity to MEK inhibitors. The MEK inhibitor cobimetinib inhibits p-ERK in both WT and RASGRP4^D404N^-expressing cells up to 48 h (Supplemental Fig. S5B). However, all cell lines were equally sensitive to growth inhibition by cobimetinib, as well as the test compound PD98059 (Supplemental Fig. S5C).

### RASGRP4 mutations confer resistance to doxorubicin

**Figure 5:**
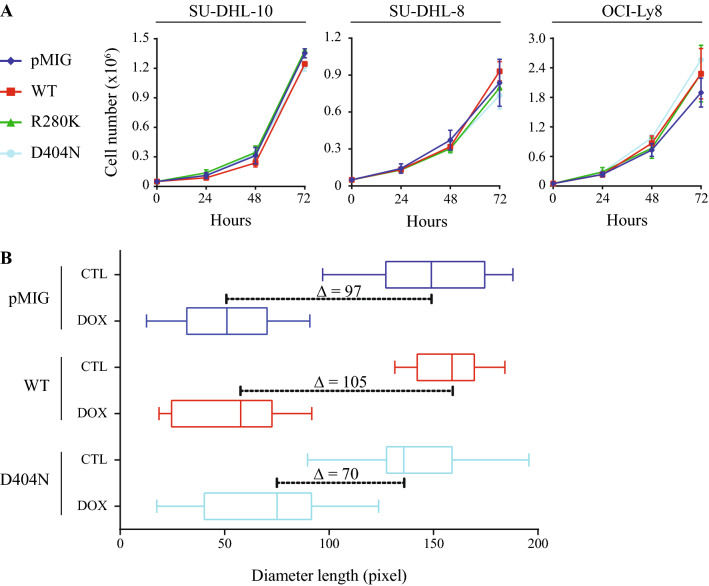
(**A**) Growth curves of stable cell lines show no difference between mutant and wild-type RASGRP4 GCB-DLBCL cell lines. Viable cell number was assessed by trypan blue staining over 3 days in culture. (**B**) SU-DHL-8 cells overexpressing mutant RASGRP4^D404N^ are more resistant than RASGRP4^WT^ cells or parental cells expressing empty vector when treated with 2.5 ng/mL doxorubicin for 12 days. A representative plot of one biological replicate is shown.

Based on the predicted increased RAS interactions and observed p-ERK, we looked at biological outcomes associated with *RASGRP4* mutation. These mutations were identified in rrDLBCL patient samples, thus we tested whether *RASGRP4* mutation changes proliferation and/or the response to chemotherapy. Expression of wild-type or mutant RASGRP4 did not change the proliferation of DLBCL cells, as assessed by trypan blue over 3 days (Fig. 5A). Next, we used a colony assay that models the selective pressure of small clones to survive and proliferate in the face of chemotherapy. Thus, we seeded SU-DHL-8 cells into wells ± 2.5 ng/mL doxorubicin for 12 days. The RASGRP4^D404N^ containing cells had a survival advantage, in the presence of doxorubicin, compared to the wild-type or vector-containing SU-DHL-8 (Fig. 5B). This indicates that there might be clonal selection for lymphoma cells harbouring *RASGRP4* mutations upon R-CHOP treatment.

## Discussion

Relapse in DLBCL after standard treatment is still considerable and the mechanisms behind R-CHOP resistance are poorly understood. We hypothesized that mutations increased in rrDLBCL samples compared to diagnostic specimens would lead to increased proliferative and/or survival signals leading to drug resistance. Indeed, this strategy has identified an increased frequency of known resistance-related genes, such as *TP53 and MYD88* (L265P), the latter of which confers resistance to granzyme B and perforin-mediated killing by CD8 + cytotoxic T cells^36^. Based on the frequency in other tumor types, we anticipated finding *RAS* and *RAF* mutations, which could provide a mechanism of resistance. While we surprisingly did not observe any *RAS* mutations, we found a number of RAS-associated proteins that were mutated. Several of these proteins have a role in other tumor types^37–41^. We hypothesized that specific RAS-associating protein mutations would correlate with the resistance to chemotherapy. This is the first report of *RASGRP4* mutations that affect its function. Using a combination of in silico and in vitro assays, we show that patient-relevant *RASGRP4* mutations enhance signaling of the RAS/MEK/ERK pathway, but not p-AKT signaling, and have a survival advantage compared to wild type after exposure to doxorubicin. Curiously, we do not observe an increased sensitivity to MEK/ERK inhibitors, suggesting that other pathways are important for potential resistance mechanisms. It is possible that a subset of the mutations found needs to be combined with other mutations or alteration to promote resistance.

Mutations in genes involved in the RAS/MEK/ERK pathway may be a feature in a subset of rrDLBCL. While *RAS* and *RAF* are commonly mutated in other cancers, this is not the case in de novo or rrDLBCL^2,16,17,22–25^. In contrast, we found mutations in multiple genes encoding RAS-associating proteins, including GEFs, GAPs, and downstream effectors, in 36% of rrDLBCL patients. No single gene was mutated at high frequency, but mutation as a collective family was enriched. This correlates with another study comparing single nucleotide variants between paired diagnostic and relapsed biopsies, where 2 of 6 tumors gained mutations in *RASSF7* at relapse^42^. Interestingly, mutations in RAS-associating proteins was more recurrent in the GCB subtype of DLBCL at relapse. *RASGRP4* mutations were found only in rrGCB-DLBCL. Compared to the ABC subtype, rrGCB-DLBCL had a higher gene expression level of RAS and RAS-associating proteins. Moreover, there was a gain of p-ERK in GCB-DLBCL at relapse. Thus, RAS signaling might have an important role in GCB-DLBCL.

Based on the increased frequency, we predicted that these mutations might lead to activation of the RAS/MEK/ERK pathway to enhance survival and proliferation of tumor cells. Indeed, we find that p-ERK expression is higher in relapsed/refractory DLBCL tumors than diagnostic. Moreover, 33% of tumors gain p-ERK expression when comparing matched relapsed and diagnostic biopsies from the same patient. Rituximab treatment leads to decreased MEK/ERK signaling in vitro by up-regulation of RKIP to ultimately decrease Bcl-X_L_ levels^43^. The RAS/MEK/ERK is well known to mediate chemo-resistance^13^. Chemoresistance is promoted not only by activating mutations in RAS/RAF/MEK, but also by activation of ERK through growth factor stimulation, interactions with extracellular matrix proteins, and endogenous signals^44^. ERK activation can promote chemoresistance by increasing adaptation of cells to ER stress induced by increasing intracellular calcium^45^ and to reactive oxygen species^46^. ERK mediates cell survival by modulation of Bcl-2 family members, increases proliferation, upregulation of drug efflux pumps, and modulation of the tumor microenvironment towards a pro-tumor phenotype^44^. However, we did not find an increased dependence on the MEK/ERK pathway for survival in our in vitro analyses when mutant RASGRP4 was expressed. Thus, p-ERK may provide a marker for R-CHOP resistance, but other RAS-mediated pathways may be responsible to the resistance phenotype. These pathways could include activation of Ca^2+^-mediated pathways^47^ and/or upregulation of anti-oxidant pathways, such as Nrf2^48,49^. In addition, mutations in RAS associating proteins may work in collaboration with each other, as 36% of samples with a RAS associating protein mutation had more than one.

We chose to further investigate the link between *RASGRP4* gene mutations and resistance, because no link to DLBCL had been described. Two closely related GEF, RASGRP1 and RASGRP3 are activated downstream of the B cell receptor, but RASGRP4 is only known to be important for mast cells, certain dendritic cells and neutrophils^29,50,51^. RASGRP4 is the GEF that mediates GPCR-stimulated RAS activation in unprimed, but not primed, neutrophils^31,52^. RASGRP4 is necessary for ERK activation, ROS production, and migration following neutrophil stimulation^31^. Aberrant activation of RASGRP4 expression is linked to rheumatoid arthritis^53,54^. Thus, these are the first data to implicate a role for RASGRP4 mutations in B cell malignancies. A previous report indicated that wild-type RASGRP4 has a role in the proliferation and survival in DLBCL cells^29^. However, we found no change in proliferation between RASGRP4-expressing cells over 3 days. In a CFU assay, cells harboring a *RASGRP4* mutation had a growth advantage compared to wild-type cells when treated with doxorubicin. These data suggest that *RASGRP4* mutation could provide a survival benefit to generate resistant-subclones. We believe these findings will help to identify suitable alternative MAPK-targeted therapies to R-CHOP in GCB-DLBCL.

## Materials and methods

### Patient data

The “relapse” cohort as well as whole exome sequencing and microarray analysis was previously described^10^. In addition, we obtained 7 additional matched diagnostic and relapsed/refractory DLBCL samples from the Leukaemia Cell Bank of Quebec-Lymphoma axis for immunohistochemistry. This project was approved by the research ethics boards at the Jewish General Hospital and is in accordance with the declaration of Helsinki.

### Immunohistochemistry

Slides containing 5 μm FFPE sections were stained for p-ERK. Slides were incubated with primary antibody for 30 min at 37℃ using an automated Ventana system (Roche). Localization of DLBCL cells was estimated by hematoxylin and eosin (H&E) stain in order to match with the p-ERK staining; > 5% of the DLBCL cells was considered positive. (See Supplemental Method for Reagents and Tools table).

### Computational study of the structural effects of *RASGRP4* mutations

The three-dimensional structure of wild-type RASGPR4 is mainly available in the Protein Data Bank (PDB ID: 6AXG). The system was prepared by using Molecular Operating Environment (MOE) software: hydrogen atoms were added and minimized, protonation states were conveniently assigned, and crystallographic waters were removed. Missing loops, i.e. residues 75–78, 109–111 and 170–194, were de novo modelled by applying the loop modeler module available in MOE. R280K and D404N RASGPR4 mutants were obtained by manually mutating the residues involved. (Supplemental Fig. S4A).

### Protein relaxation by molecular dynamics simulations

Molecular Dynamics (MD) simulations were applied using AMBER software to obtain stable conformations for wt RASGRP4 protein and its R280K and D404N mutants. Amber ff13 forcefield was used for the parameters of standard amino acids and TIP3P as water model. All systems were prepared following a gradual energy minimization protocol before starting the simulation process. The system was initially subjected to a first 5000-step of solvent energy minimization followed by a 20,000-step energy minimization of the modelled parts. After that, the entire molecular system was energy minimized with a 20,000-steps long simulation without constrains.

After minimization, the system was heated to 300 K within 200 ps using the Langevin thermostat. Pressure equilibration (1 bar) was performed for 1000 ps under a NPT ensemble. The production phase was extended to 20 ns defining a 2 fs time-step. The SHAKE algorithm was used throughout to restrain the bonds involving hydrogens and the Particle Mesh Ewald method for long range electrostatic, while the short-range interactions had a 10 Å cutoff radius. (Supplemental Fig. S4B^55,56^).

### Protein–protein docking

Protein–protein docking was performed using MOE to predict the interaction mechanism between HRas and wt and mutated RASGPR4 proteins. The most representative structures of RASGRP4 proteins was obtained by cluster analysis applied on the 20 ns MD. Dockings were performed using HRas as ligand. Considering the data available in the 6AXG PDB entry, it contains an incomplete three-dimensional structure of HRas (two loops, i.e. 27–32 and 118–121, were modelled by homology modelling). Hydrophobic patch potential analysis was used to guide the blind docking procedure. (Supplemental Fig. S4C).

Protein–protein docking protocol was validated by comparing the computational results with crystallographic data, available for the wild-type protein. Results indicate that the protein–protein docking was able to recognize the active site described in the literature for the wild-type protein^33,57^. The best ranked conformation of the HRas with modelled loops perfectly fits the interacting conformation described in the 6AXG PDB (RMSD = 0.96A). (Supplemental Fig. S4D).

MD simulations were applied in order to evaluate the dynamic behavior of the best ranked complexes. The same protocol as the protein relaxation MD was used defining a 100 ns production stage. (Supplemental Fig. S4E).

The study of the changes on the hydrogen bond pattern was performed using CPPTRAJ. Hydrogen bonds are determined using simple geometric criteria such as the donor to acceptor heavy atom distance.

### MMPBSA calculations

The binding free energy for the association of both proteins was calculated using the Molecular Mechanics Poison-Boltzmann Surface Area (MM-BPSA) methodology available in AMBER 14.

Waters and counted ions were removed. For each complex, 2000 conformations were extracted from the last 20 ns of the MD simulations. The interaction energy and solvation free energy for complex, receptor, and ligand were calculated to estimate of the binding free energy.

### Cell line culture

Phoenix AMPHO cells [CRL-3213; American Type Culture Collection (ATCC)] were grown in DMEM media supplemented with 10% fetal bovine serum (FBS). OCI-Ly8, SU-DHL-8, and SU-DHL-10 GCB-DLBCL cell lines were cultured in RPMI containing 10% FBS. OCI-Ly8 cells were a gift from Dr. Riccardo Dalla Favera (Columbia University) and SU-DHL-10 and SU-CHL10 were purchased from ATCC. All cell line identities were confirmed via short tandem repeat (STR) testing (The Centre for Applied Genomics, Toronto, Canada). Growth curves were assessed by plating 50,000 cells and counting viable cells every day using Trypan blue exclusion.

### Cloning and site-directed mutagenesis

The pcDNA3.1-h*RASGRP4* was provided by Dr. Takao Koike (Hokkaido University Graduate School of Medicine)^53^. The coding sequence of human *RASGRP4* (NM_170604.3) was amplified by PCR (See Supplemental Method for primer sequences) and digested with the restriction enzymes EcoRI and XbaI. The insert was ligated to the p3xFLAG-CMV-14 vector. By site-directed mutagenesis, we generated three h*RASGRP4*-3xFLAG (C-terminal tag) mutated constructs (R280K, R304R and D404N) in the p3xFLAG-CMV-14 vector. Subsequently, the wild-type and mutated h*RASGRP4*-3xFLAG inserts were amplified by PCR and cut with the restriction enzymes EcoRI and XhoI. The inserts were ligated into the pMSCV-IRES-GFP (pMIG) vector.

### Generation of transient and stable RASGRP4 mutant cell lines

Phoenix-AMPHO cells were transfected with p3xFLAG-CMV-14 plasmids containing wild-type or mutant h*RASGRP4* to generate transiently-expressing cell lines. To produce stable cell lines with retrovirus, Phoenix-AMPHO packaging cells were transfected with the pMIG plasmids containing wild-type or mutant h*RASGRP4*. Subsequently, the OCI-Ly8, SU-DHL-8 and SU-DHL-10 GCB-DLBCL cell lines were transduced by spinoculation (centrifugation at 800G for 30 min at room temperature) with the retrovirus every 12 h for two consecutive days. The GFP^+^ cells were sorted with a BD FACSAria Fusion Cell Sorter at the Lady Davis Institute Flow Cytometry Facility to generate stable cell lines.

### Western blotting

The level of p-ERK in transduced GCB-DLBCL cell lines were assessed in normal serum conditions, following stimulation with or without phorbol 12-myristate 13-acetate (PMA) (100 nM) for 30 min in low serum-containing media (1% FBS) overnight. Cells were lysed in RIPA buffer and sonicated for 10 s. Twenty ug of protein, except when forty ug was needed for p-ERK and p-AKT, were separated using 10% SDS-PAGE. The gels were transferred on nitrocellulose membrane, blocked with 5% milk tris buffer saline for 1 h and were incubated with primary antibody overnight at 4℃ followed by secondary antibody for 2 h at room temperature. The nitrocellulose membranes were incubated for 1 min with ECL and exposed on films (exposure time range: 1 s. to 5 min.).

### Colony forming unit (CFU) assay

Five SU-DHL-8 cells were seeded in U-shaped 96-wells plates with 25% conditioning media (from the SU-DHL-8-pMIG cells) and treated with 2.5 ng/mL doxorubicin. At day 4 and 8, half of the media was changed, and the cells were re-treated with 2.5 ng/mL doxorubicin. The diameter of the colony in each well was evaluated at day 12, a time point that allowed vehicle-treated cultures to reach 10,000 cells per well, as based on their doubling time. A total of twenty wells (technical replicate) were seeded per condition and the experiment was repeated at least 3 times (biological replicate).

### Statistical analyses

Data was analyzed by two-tailed χ^2^ test, Student’s *t*-test (unpaired, two-tailed), or one-way ANOVA with Tukey’s post-hoc analysis.

## Supplementary Information

Supplementary Information 1.

Supplementary Information 2.

Supplementary Information 3.

## Abbreviations

ABC: Activated B-cell
COO: Cell of origin
CFU: Colony forming unit
DLBCL: Diffuse large B cell lymphoma
GAP: GTPase-activating protein
GCB: Germinal center B-cell
GEF: Guanyl exchange factor
MD: Molecular dynamics
MOE: Molecular operating environment
PMA: Phorbol 12-myristate 13-acetate
pMIG: PMSCV-IRES-GFP
PTM: Post-translational modification
RASGRP4: RAS guanyl releasing protein 4
rrDLBCL: Relapse/refractory DLBCL
TLy: Transformed follicular lymphoma
