# Supplementary material for: Mutated RAS-associating proteins and ERK activation in relapse/refractory diffuse large B cell lymphoma
Source: Sci Rep. 2022 Jan 17;12:779. doi: 10.1038/s41598-021-04736-0 (PMC8764096; doi:10.1038/s41598-021-04736-0)

Mutated RAS-associating proteins and ERK activation in relapse/refractory Diffuse Large B cell lymphoma

Alexandre Benoit[1,2], Elisabeth Bou-Petit[3], Hsiang Chou[1,2], Melissa Lu[4], Cynthia Guilbert[1], Vincent Mingyi Luo[1,5], Sarit Assouline[1,2,6], Ryan D. Morin[7], Svetlana Dmitrienko[8], Roger Estrada-Tejedor[3], Nathalie A. Johnson[1,2,6], Koren K. Mann[1,2,6*]

Figure S1. Schematic representation of the mutations found in Ras-associating proteins in rrDLBCL patients.

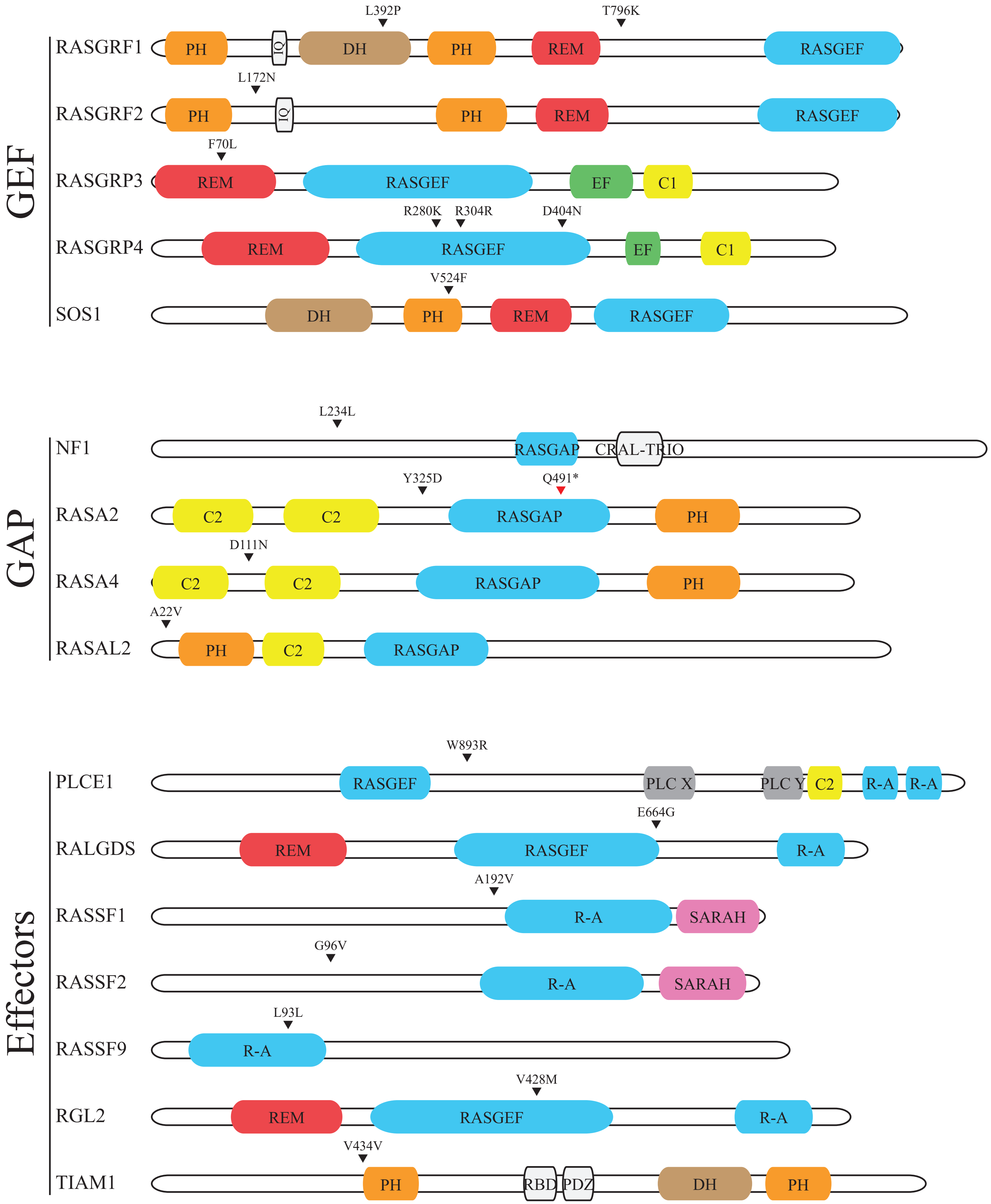

Figure S2. Gene expression of RAS and RAS-associating proteins in rrDLBCL patient biospsies. A) mRNA expression levels of post-translational modification (PTM) enzymes associated with the RAS pathway are higher in rrGCB-DLBCL samples compared to rrABC-DLBCL samples. B) RAS-associating protein mRNA expression levels are not altered between samples with or without a RAS-associated protein mutation with the exception of PIK3CD. (unpaired t-test; * = p-value < 0.05, ** = p-value < 0.01)

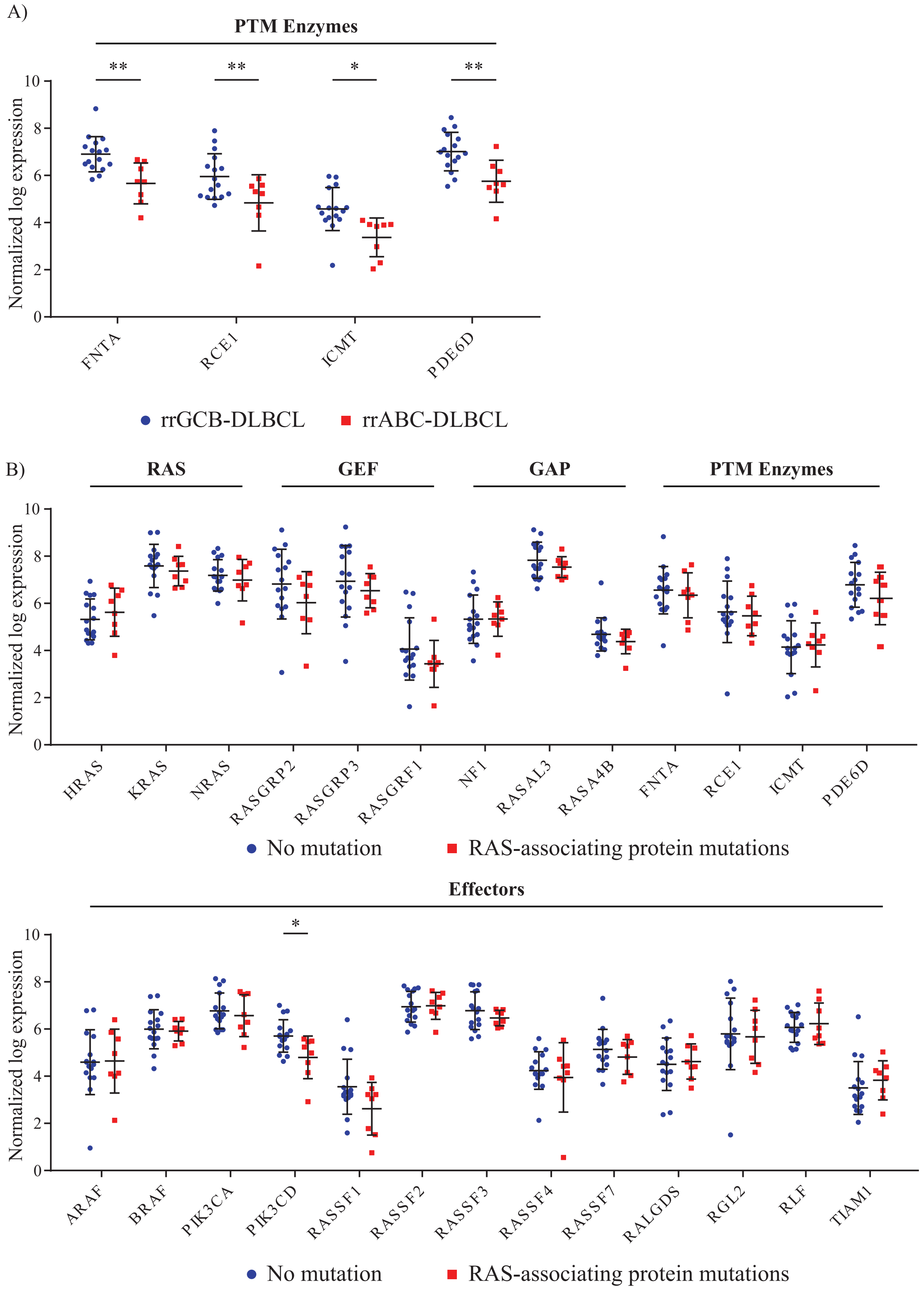

Figure S3. Sequence alignment of human RASGRP family members and RASGRP4 amongst different species shows conservation of D404 amino acid. Only RASGRP4 has an arginine at position R280 when compared to the other RASGRP family member and it is not conserved among different species.

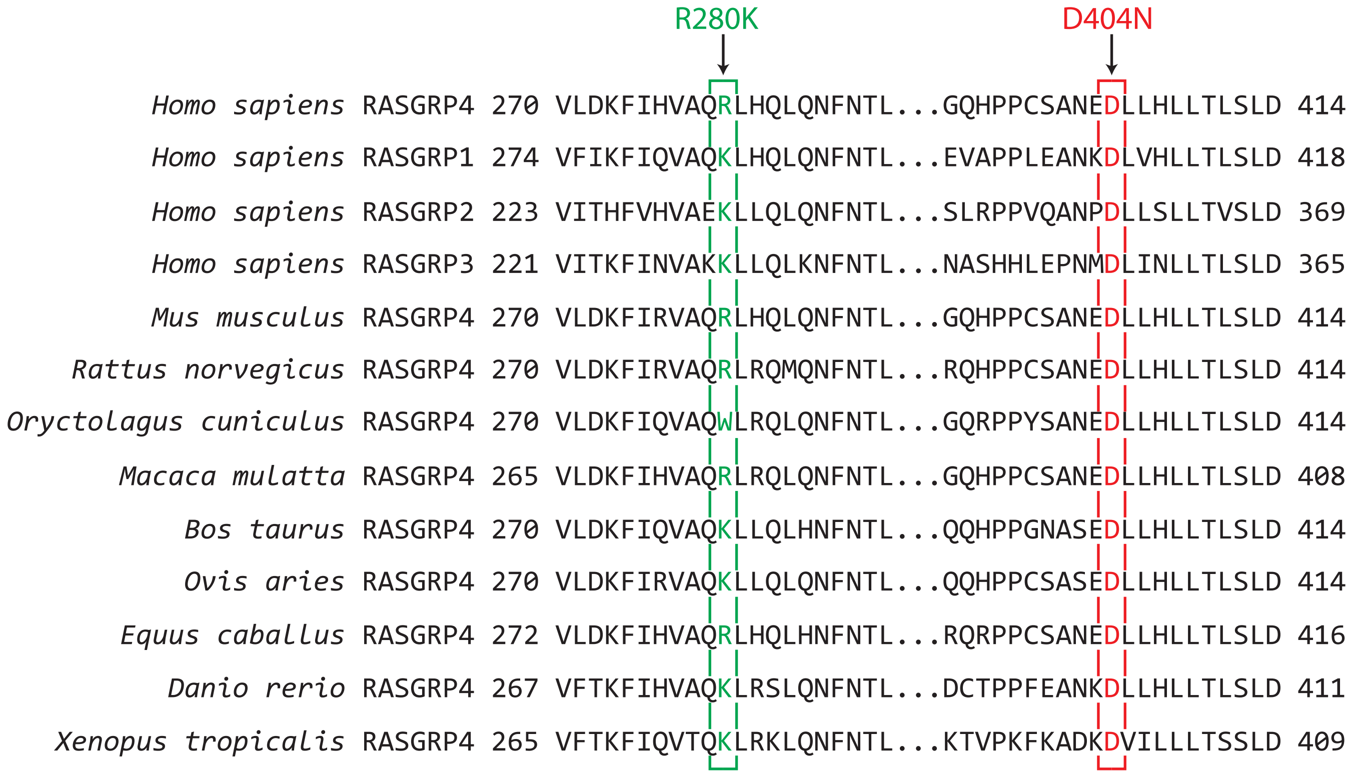

Figure S4. Molecular Modeling of HRas and RASGRP4. A) Crystal structure of RASGRP4 (green) in complex with HRas (red) (PDB ID 6AXG). Yellow spots mark the position of the mutations under study. Missing parts (black) are modelled to conduct the study. B) RMSD of the 20 ns MD simulation performed for the refinement of homology models of RASGRP4 wild-type, R280K, and D404N mutants. RMSD is calculated considering all heavy atoms. C) Graphical representation of the best ranked conformations obtained from the guided protein-protein docking. The interaction area of HRas was defined considering the same residues reported in the 6AXG PDB file. RASGRP4 and HRas (red) were considered as rigid bodies in all calculations. A total of 500 complex conformations were evaluated and energy minimized to identify the best pose. D) Validation of the docking parameters. Comparison between the position of predicted HRas (red) and its experimentally observed conformation (green). HRas was docked in the incomplete RASGRP4 structure of the PDB. RMSD between the predicted structure and the crystalized structure is 0.96Å. E) RMSD of the RASGRP4-HRas complexes during 100 ns MD simulation. RMSD was calculated considering all heavy atoms of the RASGPR4 protein. Only the last 20 ns of each simulations were used to estimate the binding energy through MMPBSA.

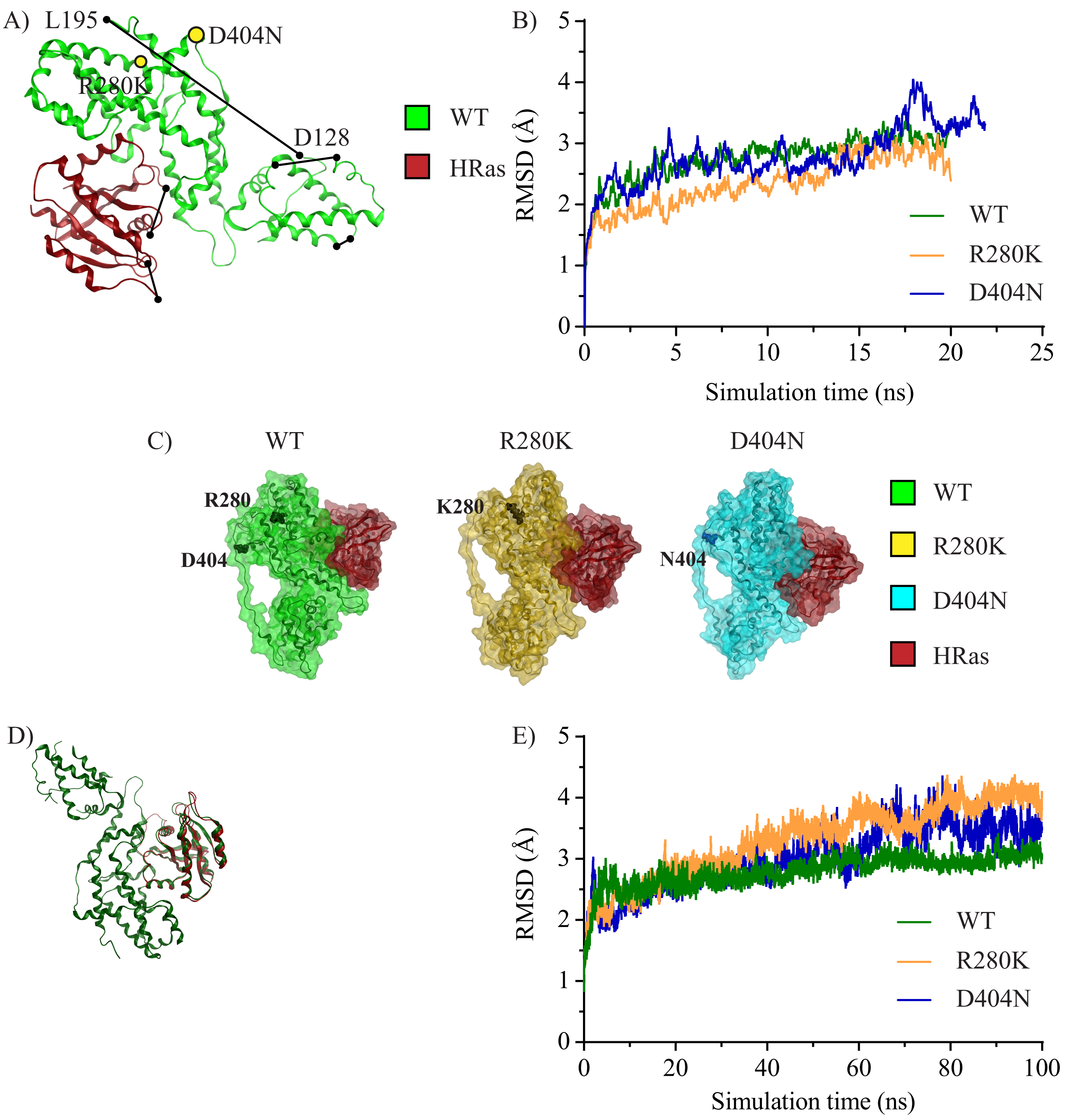

Figure S5. RASGRP4 mutation does not alter PMA-induced ERK phosphorylation or sensitivity to MEK inhibitors. A) Stimulation with 100 nM phorbol 12-myristate 13-acetate (PMA) for 30 minutes in low serum condition (1% FBS, overnight) shows that mutant RasGRP4D404N cells have a higher level of p-ERK than RasGRP4WT cells in OCI-Ly8, but not other cell lines. B) Cobimetinib (1 μM) inhibits p-ERK in SU-DHL-8 cells expressing pMIG vector, RasGRP4WT, or RasGRP4D404N up to 48 hours following exposure. C) SU-DHL-8 cells were cultured in the presence of varying concentrations of cobimetinib or PD98059 for 3 days and MTT performed to determine relative cell number.

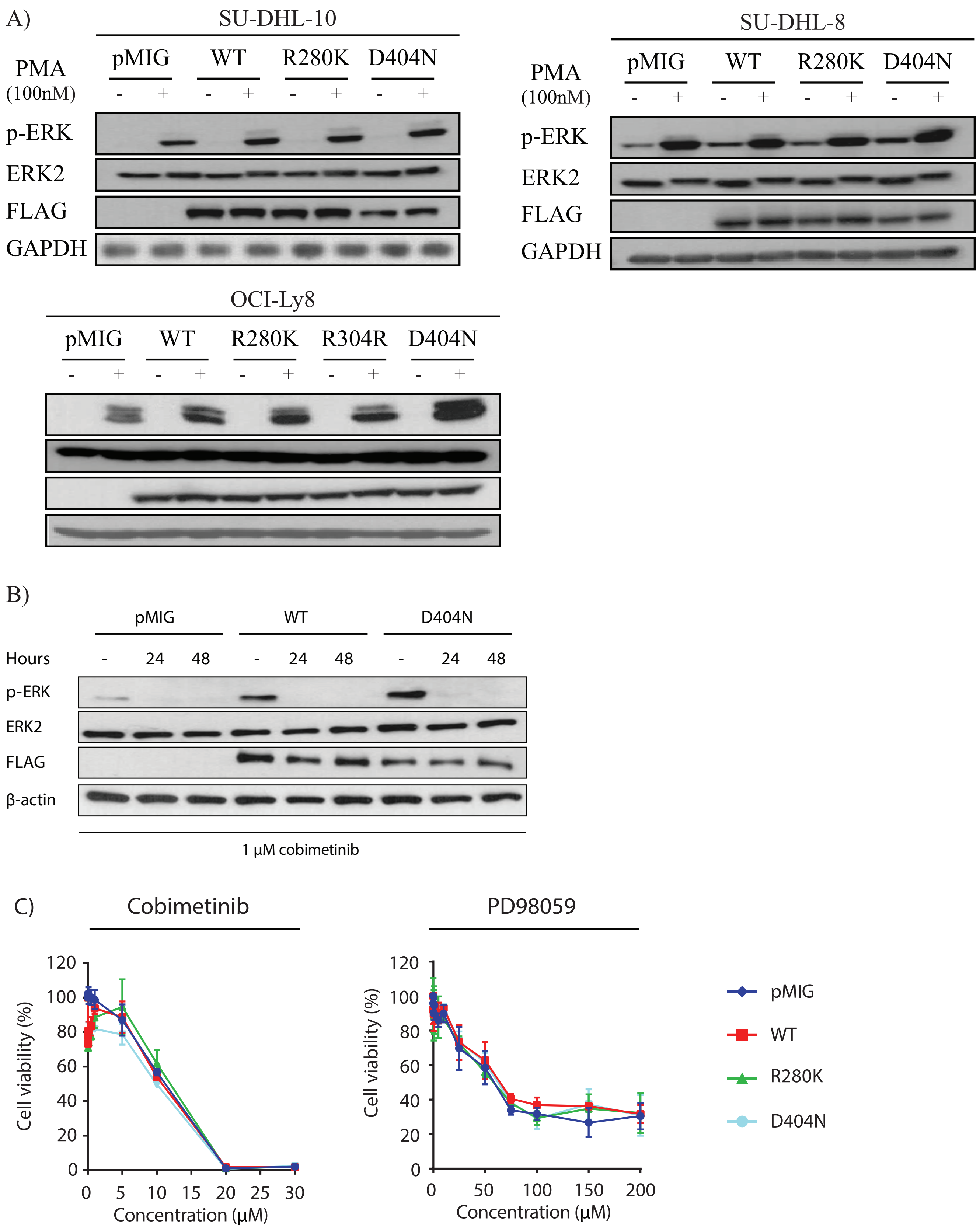

Supplement: Supplementary file 1 — Supplementary Information 1. [file 41598_2021_4736_MOESM1_ESM.pdf]
