# Supplementary material for: Mutated RAS-associating proteins and ERK activation in relapse/refractory diffuse large B cell lymphoma
Source: Sci Rep. 2022 Jan 17;12:779. doi: 10.1038/s41598-021-04736-0 (PMC8764096; doi:10.1038/s41598-021-04736-0)
Supplement: Supplementary file 2 — Supplementary Information 2. [file 41598_2021_4736_MOESM2_ESM.pdf]

SUPPLEMENTAL METHODS

Reagents and tools table

Reagent or resource	Reference or source	Identifier or catalog number
**Experimental models**		
OCI-Ly8	Dr. Riccardo Dalla-Favera	N/A
SU-DHL-8	ATCC	CRL-2961
SU-DHL-10	ATCC	CRL-2963
Phoenix AMPHO	ATCC	CRL-3213
**Recombinant DNA**		
pcDNA3.1-hRASGRP4	Hashimoto et al, 2011	N/A
p3xFLAG-CMV-14	Sigma	E7908
p3xFLAG-CMV-14-hRASGRP4	This paper	N/A
pMSCV-IRES-GFP	Addgene	20672
pMSCV-IRES-GFP-hRASGRP4-3xFLAG	This paper	N/A
**Antibodies**		
Phospho-p44/42 MAPK (Erk1/2) (Thr202/Tyr204) Rabbit monoclonal (1:100; IHC)	Cell Signaling	4376
GAPDH Rabbit monoclonal (1:1000)	Cell Signaling	2118
Phospho-p44/42 MAPK (Erk1/2) (Thr202/Tyr204) Rabbit polyclonal (1:1000; W.B.)	Cell Signaling	9101
Phospho-Akt (Ser473) Rabbit polyclonal (1:1000)	Cell Signaling	9271
FLAG-M2 Mouse monoclonal (1:5000)	Sigma-Aldrich	F3165
β-actin Mouse monoclonal (1:10,000)	Sigma-Aldrich	A5441
ERK2 Rabbit polyclonal (1:500)	Santa Cruz Biotechnology	sc-154
AKT1 Rabbit polyclonal (1:500)	Santa Cruz Biotechnology	sc-1618-R
Rabbit IgG HRP Linked Whole Ab (1:3000)	GE Healthcare	NA934
Mouse IgG HRP Linked Whole Ab (1:5000)	GE Healthcare	NA931
**Oligonucleotides and other sequence-based reagents**		
PCR primers	This study	Table EV1
**Chemicals, Enzymes and other reagents**		
EcoRI	Roche	10606189001
XbaI	Roche	11047663001
XhoI	Roche	11047663001
T4 DNA Ligase	NEB	M0202
Protease Inhibitor Cocktail	Roche	04693116001
PhosphoStop Inhibitor Cocktail	Roche	04906837001
ECL chemiluminescent detection reagent	GE Healthcare	RPN2106

phorbol 12-myristate 13-acetate	Sigma	P1585
Goat Anti-Human IgM-UNLB	Southern Biotech	2020-01
doxorubicin	Pharmacy of the Jewish General Hospital	N/A
**Software**		
Prism (version 6.0)	GraphPad	
Aperio Image Scope (version 12.3.3)	Leica Bio system https://www.leicabiosystems.com/digital-pathology/manage/aperio-imagescope/	
Illustrator (CS6)	Adobe	
ImageJ (version 2)	NIH https://imagej.net/Welcome	
PolyPhen-2 (version 2.2)	Adzhubei et al, 2010 http://genetics.bwh.harvard.edu/pph2/	
Molecular Operating Environment (MOE) (version 2016.08)	Chemical Computing Group https://www.chemcomp.com/Products.htm	
AMBER (version 14)	https://ambermd.org/	
**Other**		
Ventana system	Roche	
BD FACSAria Fusion Cell Sorter	BD Bioscience	
Gelcount Plate Scanner	Oxford-Optronix	

Primer sequences for PCR amplification and site-directed mutagenesis

hRasGRP4-EcoRI site - Forward

GCGAATTCAAGCATGAACAGAAAAGACA

hRasGRP4-XbaI site - Reverse

GCTCTAGAGGAATCCAGCTTGGA

hRasGRP4-3xFLAG-XhoI site - Reverse

GCCTCGAGTGTCTACTTGTCATCGTCATCC

R280K – Forward

AGTTCATTCACGTGGCACAGAAGCTCC

R280K - Reverse

GCTGGTGGAGCTTCTGTGCCACGTGA

R304R - Forward

ATGGGAGTCCTTGAGCCTGGAGATGG

R304R - Reverse

CAGTGCCATCTCCAGGCTCAAGGACT

D404N - Forward

GCAGCGCCAATGAGAATCTGCTGCAC

D404N - Reverse

CAGGTGCAGCAGATTCTCATTGGCGCT

Supplementary Table 1. RAS Associating Protein mutations identified in diagnostic biopsies.

	Percentage	Number of Cases (out of 1295)
**RASGRF1**	0.30%	4
**RASGRF2**	0.40%	5
**RASGRP3**	0.20%	2
**RASGRP4**	0%	0
**SOS1**	0.20%	3
**NF1**	3%	39
**RASA2**	0.40%	5
**RASA4**	0.20%	2
**RASAL2**	0.50%	7
**PLCE1**	0.50%	7
**RALGDS**	0.20%	3
**RASSF1**	0.20%	3
**RASSF2**	0.20%	2
**RASSF9**	0.50%	7
**RGL2**	0.10%	1
**TIAM1**	0.80%	10
