# Supplementary material for: Mutated RAS-associating proteins and ERK activation in relapse/refractory diffuse large B cell lymphoma
Source: Sci Rep. 2022 Jan 17;12:779. doi: 10.1038/s41598-021-04736-0 (PMC8764096; doi:10.1038/s41598-021-04736-0)

Mutated RAS-associating proteins and ERK activation in relapse/refractory Diffuse Large B cell lymphoma

Alexandre Benoit[1,2], Elisabeth Bou-Petit[3], Hsiang Chou[1,2], Melissa Lu[4], Cynthia Guilbert[1], Vincent Mingyi Luo[1,5], Sarit Assouline[1,2,6], Ryan D. Morin[7], Svetlana Dmitrienko[8], Roger Estrada-Tejedor[3], Nathalie A. Johnson[1,2,6], Koren K. Mann[1,2,6*]

Scans for Figure 4A: Phoenix Ampho

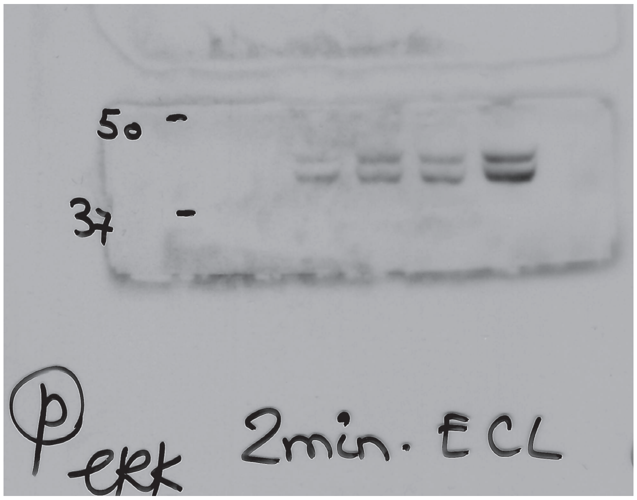

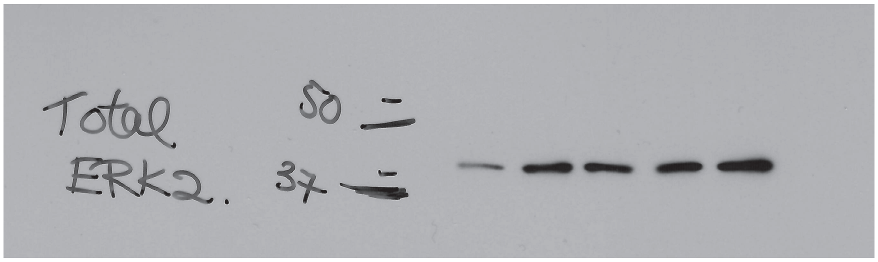

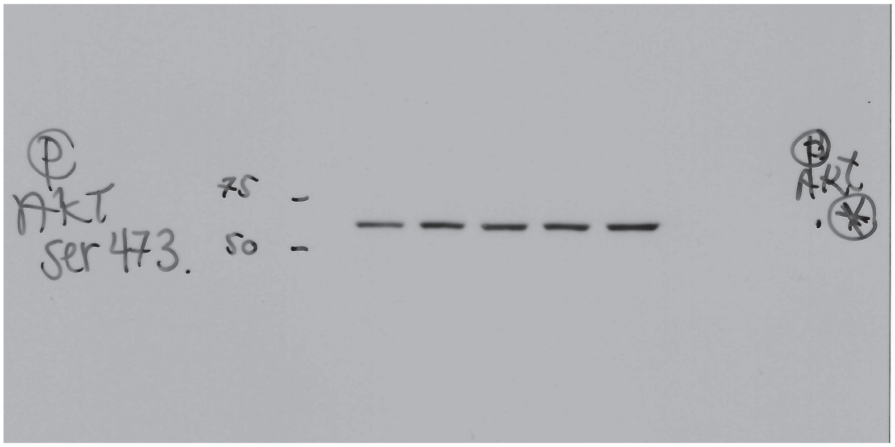

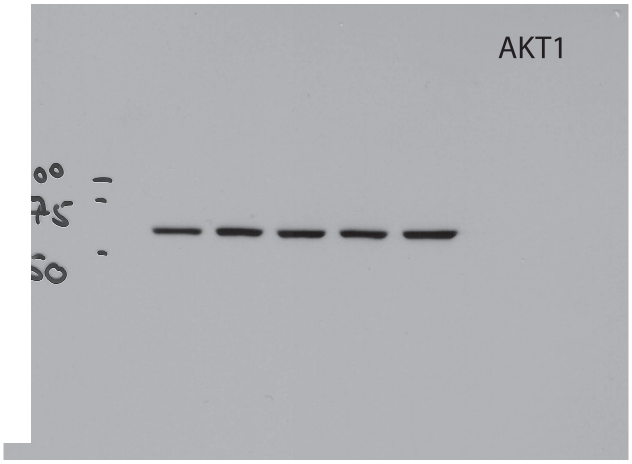

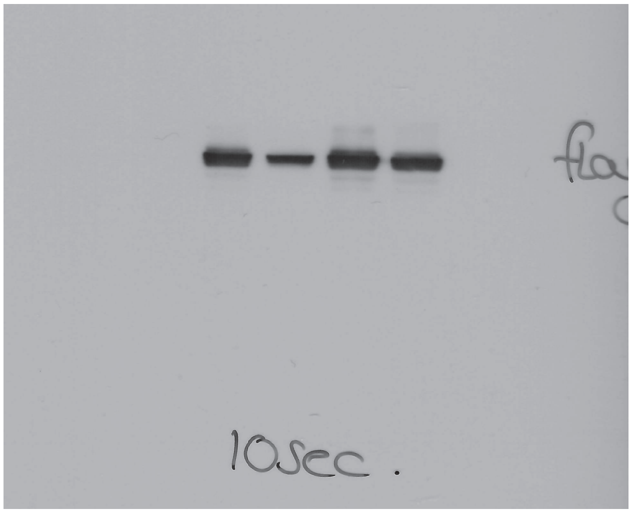

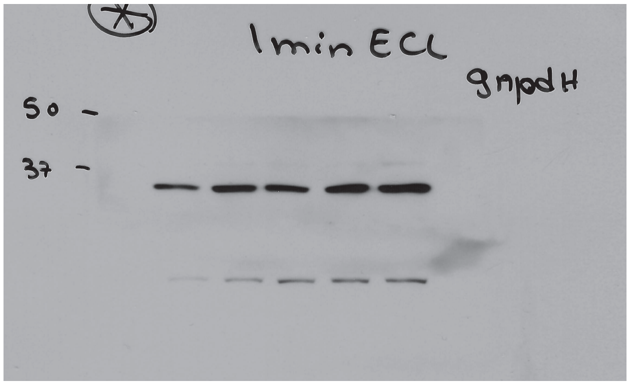

Scans for Figure 4

Figure 4B: SU-DHL8

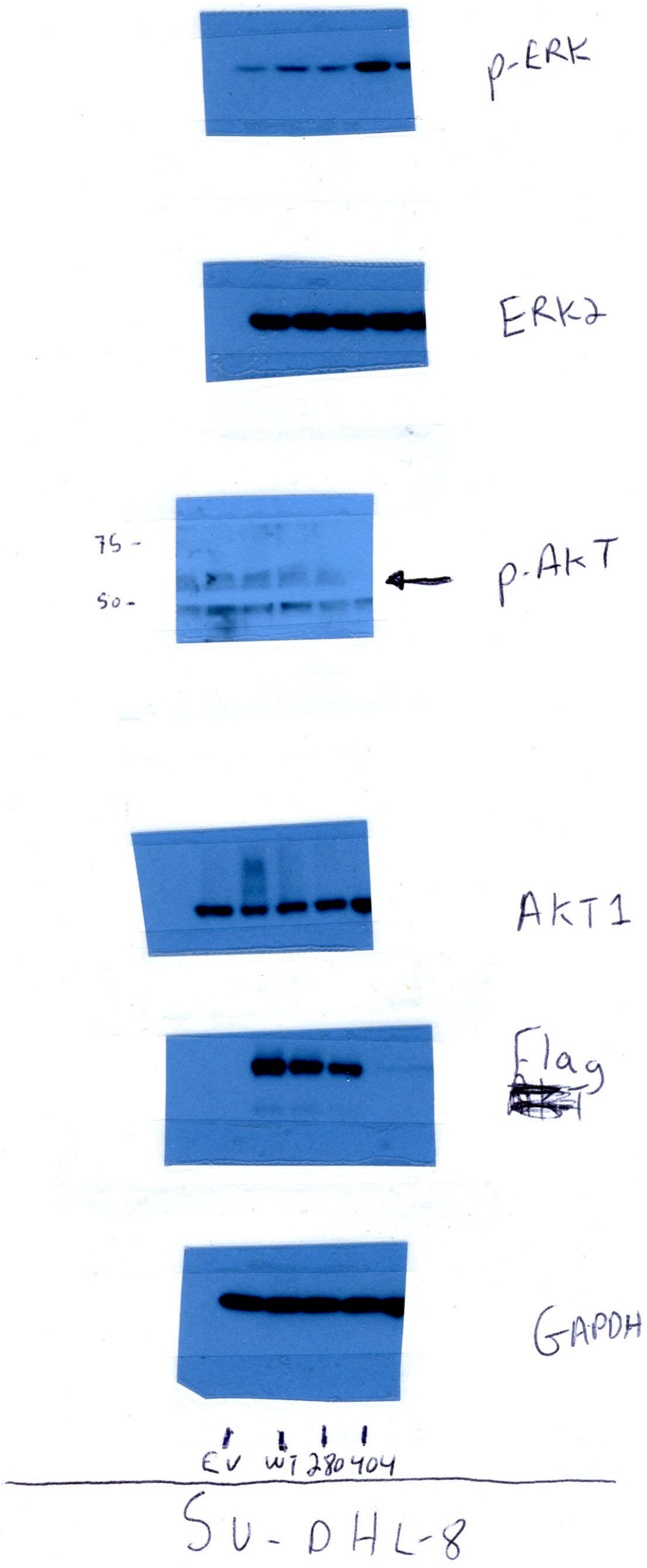

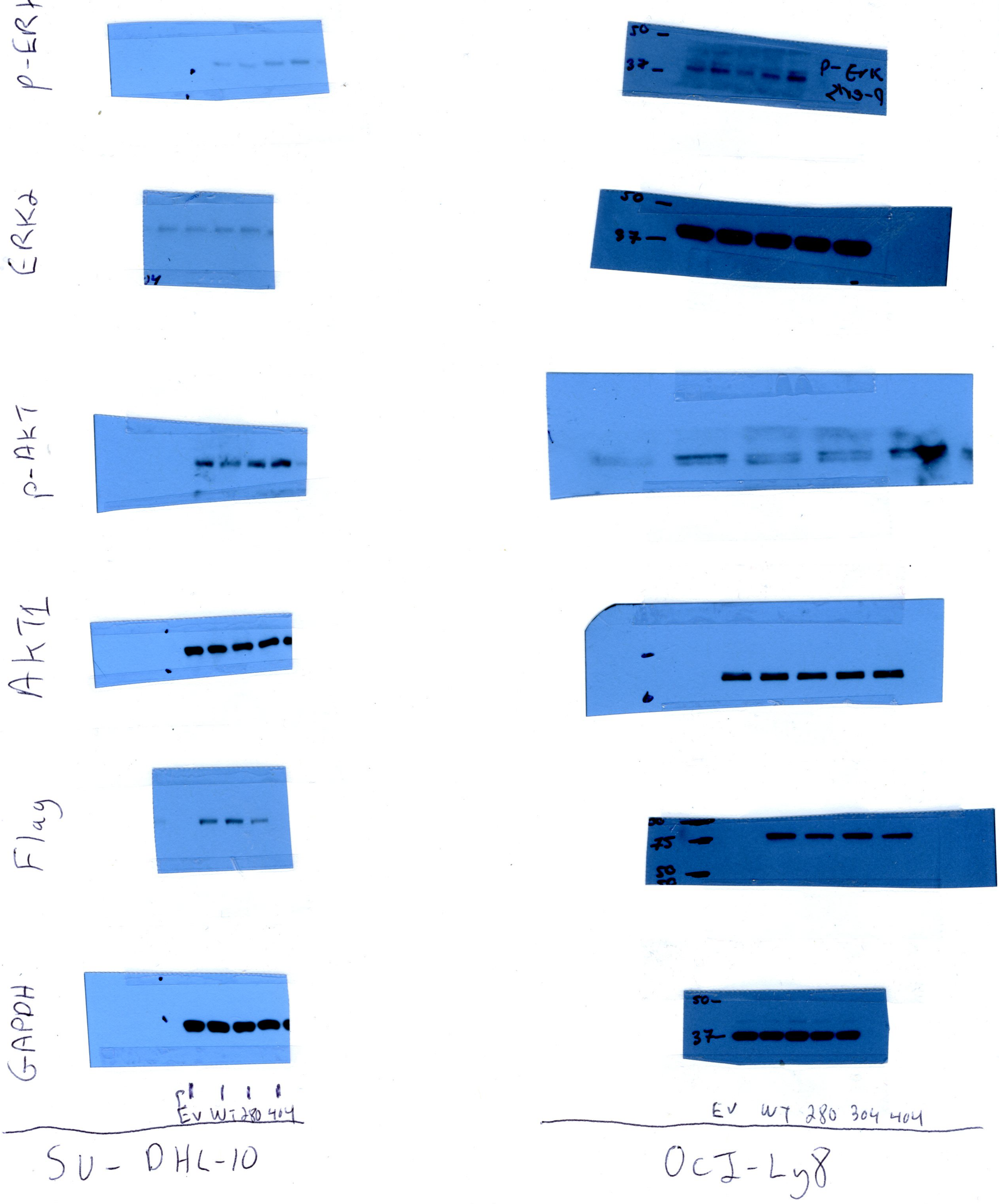

Scans for Figure 4

Figure S5 Original Scans: SU-DHL10

SU-DHL-10

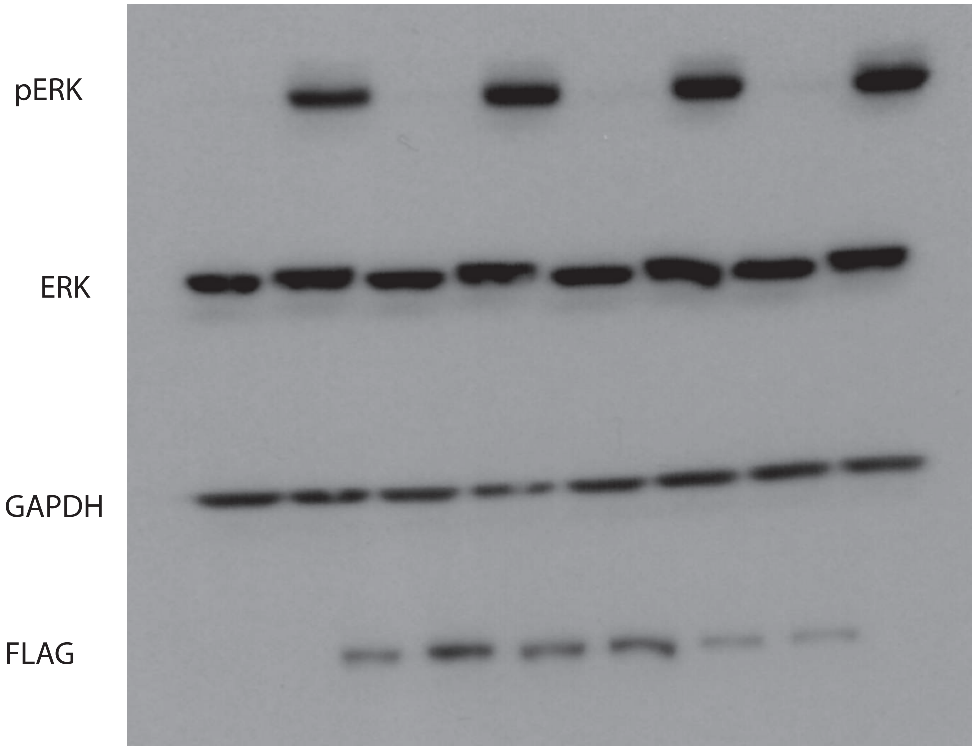

Figure S5 Original Scans: SU-DHL8 and OCI-LY8

SU-DHL8

pERK

ERK

GAPDH

FLAG

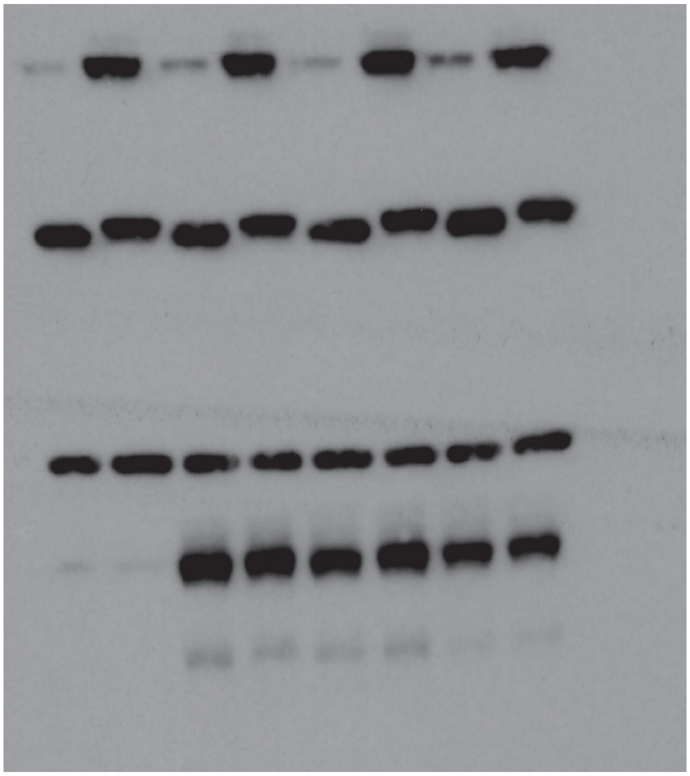

OCI-LY8

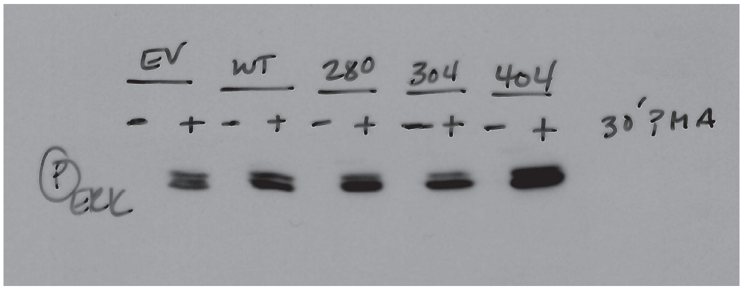

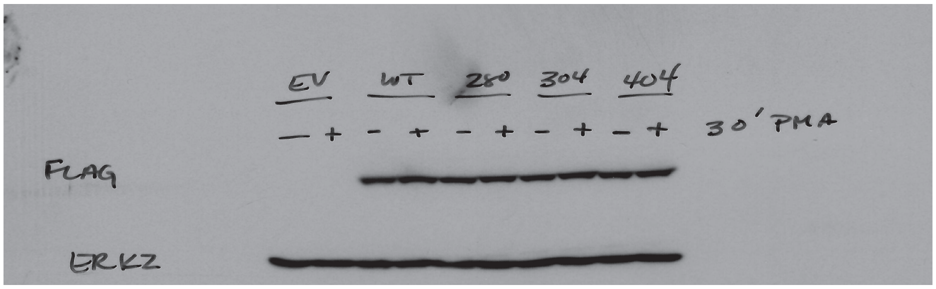

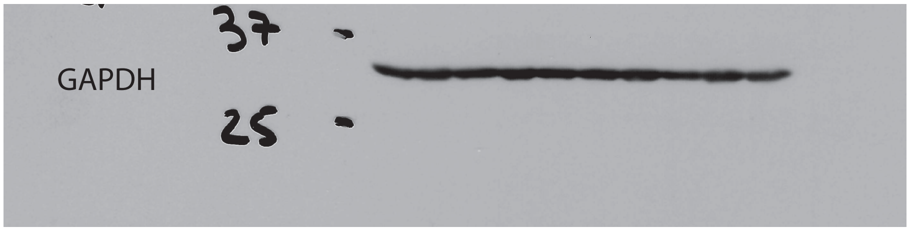

Figure S5.C: Cobimetinib

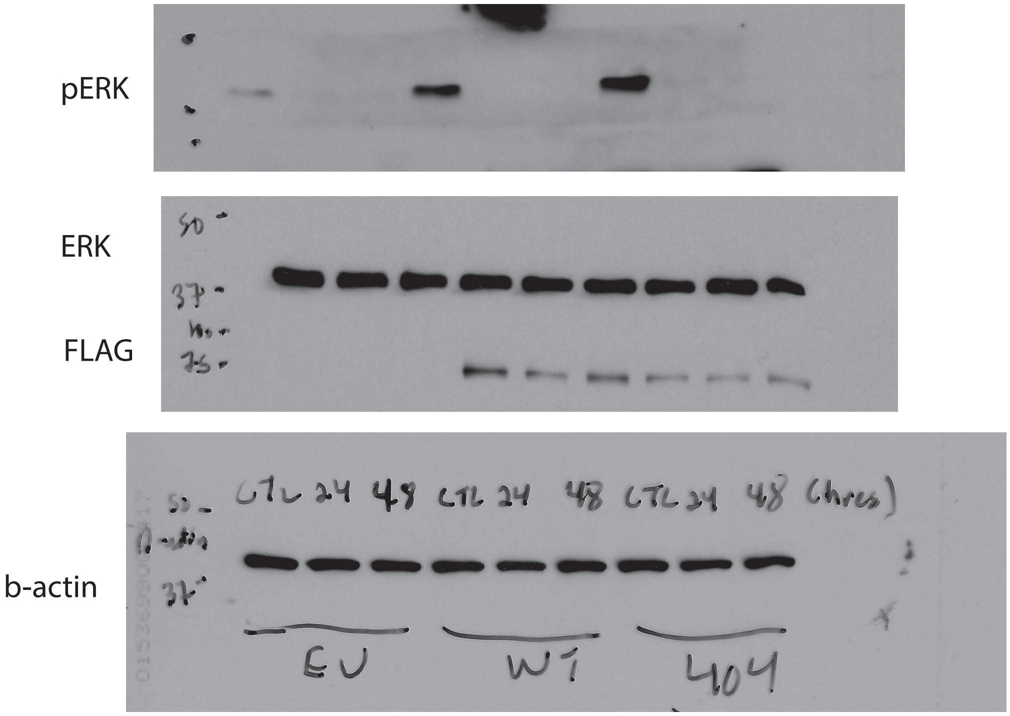

Supplement: Supplementary file 3 — Supplementary Information 3. [file 41598_2021_4736_MOESM3_ESM.pdf]
